# A chart review of substance use screening and related documentation among adolescents in outpatient pediatric clinics: implications for practice

**DOI:** 10.1186/s13011-020-00276-4

**Published:** 2020-05-25

**Authors:** Lisa M. Kuhns, Brookley Carlino, Katie Greeley, Abigail L. Muldoon, Niranjan Karnik, Hale Thompson, Robert Garofalo, Maria Rahmandar

**Affiliations:** 1 The Potocsnak Family Division of Adolescent & Young Adult Medicine, Ann & Robert H. Lurie Children’s Hospital, 225 E. Chicago Avenue, Box 161, Chicago, IL 60611 USA; 2 Department of Pediatrics, Northwestern University, Feinberg School of Medicine, Chicago, IL USA; 3 Department of Psychiatry & Behavioral Sciences, Rush Medical College, Rush University, Chicago, IL USA

**Keywords:** Drug and alcohol screening, Substance-related disorders, Adolescent, Primary care

## Abstract

**Background:**

Despite recent reductions, youth substance use continues to be a concern in the United States. Structured primary care substance use screening among adolescents is recommended, but not widely implemented. The purpose of this study was to describe the distribution and characteristics of adolescent substance use screening in outpatient clinics in a large academic medical center and assess related factors (i.e., patient age, race/ethnicity, gender, and insurance type) to inform and improve the quality of substance use screening in practice.

**Methods:**

We abstracted a random sample of 127 records of patients aged 12–17 and coded clinical notes (e.g., converted open-ended notes to discrete values) to describe screening cases and related characteristics (e.g., which substances screened, how screened). We then analyzed descriptive patterns within the data to calculate screening rates, characteristics of screening, and used multiple logistic regression to identify related factors.

**Results:**

Among 127 records, rates of screening by providers were 72% (each) for common substances (alcohol, marijuana, tobacco). The primary method of screening was use of clinical mnemonic cues rather than standardized screening tools. A total of 6% of patients reported substance use during screening. Older age and racial/ethnic minority status were associated with provider screening in multiple logistic regression models.

**Conclusions:**

Despite recommendations, low rates of structured screening in primary care persist. Failure to use a standardized screening tool may contribute to low screening rates and biased screening. These findings may be used to inform implementation of standardized and structured screening in the clinical environment.

**Clinical trial registration:**

not applicable.

## Introduction

Alcohol, drug and other substance use is a key concern for providers of adolescent medical and behavioral health care in the United States (US). Despite overall reductions in youth substance use over the past 5 years, opioids, marijuana and binged alcohol continue to see sustained use in adolescent and young adult populations [1]. In addition, while cigarette smoking continues to decline among adolescents in recent years, e-cigarette use or vaping of nicotine, marijuana and/or flavorings has increased dramatically [1]. This is a particular concern because compared to other age groups, adolescents are at particularly high risk for substance use-related health problems [2]. Substance use among teens is associated with other risk behavior and related morbidity, including teenage pregnancy, sexually transmitted infections, and domestic violence, [3] as well as social and legal issues related to substance abuse including criminal behavior, school failure, and family problems [4]. Furthermore, the leading cause of mortality between youth aged 10–24 years old is unintentional injury, and substance use increases this risk [5]. Beyond the immediate implications of adolescent substance use, early drug use is a predictor of future addiction as well as long-term sequalae [6, 7]. While undergoing crucial periods of development, the adolescent brain is particularly vulnerable to developing substance use disorders [8] and substance abuse has the potential to trigger long-term neurocognitive changes in adolescents.

Because of the unique vulnerability of adolescents and the numerous medical, social, and cognitive effects of early initiation of substance use, pediatricians are in the unique position to intervene on a pattern of behavior that could affect their patients’ lives beyond adolescence. For example, most adolescents are seen in primary care once a year, may have an on-going and trusting relationship with their provider, and often view their provider as knowledgeable on substance use and other sensitive issues, all of which provide opportunity for intervention [2].

Screening for substance use is often the first step in identifying substance use problems in adolescents. Screening is the process of asking structured questions (not just asking about substance use informally) that objectively identify those patients who are at the highest risk of substance misuse and dependence. Prior research suggests that clinician perception alone is not accurate in determining the level of substance use, and that the use of a standardized and validated screening protocol results in higher detection rates [4, 9]. Furthermore, prior research suggests that relying on provider impressions, rather than a structured screening tool, may also lead to biased screening. For example, evidence suggest physicians are more likely to screen boys than girls, and screen older adolescents versus younger adolescents [10]. To provide guidance on screening for substance use in primary care, the American Academy of Pediatrics (AAP) issued a policy statement in 2011 (revised in 2016) detailing the pediatrician’s role in decreasing the burden of substance use among adolescents [2, 11]. The AAP endorses the use of Substance Use Screening, Brief Intervention, and Referral to Treatment (SBIRT) as a method to systematically address teen drug and alcohol use [2, 11].. In adults, SBIRT has been shown to be effective in reducing alcohol and drug use [12, 13] and is backed by the U.S. Preventative Services Task Force (USPSTF) for this purpose [14]. In the adolescent population, the base of evidence is still evolving and thus, an important area for further research.

In practice, between 50 and 86% [2] of pediatricians report screening adolescent patients for substance use and often use psychosocial mnemonic tools such as the HEEADSSS (home, education/employment, eating, activities, drugs, sexuality, suicide/depression, safety from injury/violence) or SSHADESS (strengths, school, home, activities, drugs/substance use, emotions/eating/depression, sexuality, safety) as a primary framework [15]. In these mnemonics, the “D” (i.e., “drugs” or “drugs/substance use”) is a cue to the physician to ask about substance use, with a question such as “Do you use tobacco? Alcohol? Other Drugs?” [16] If an adolescent answers in the affirmative, additional structured tools should be used to assess the quantity, frequency and potential problem behavior associated with use of each substance [17]. The AAP endorses the use of a validated and age-appropriate screening tool, such as the CRAFFT, Screening to Brief Intervention (S2BI) or Brief Screener for Tobacco, Alcohol and Other Drugs (BSTAD) [2, 11]. By determining where a patient falls on the spectrum of misuse, a clinician can appropriately direct the next steps in care. For example, for patients who are not using substances, this offers an opportunity to reinforce healthy behavior. For those with low or intermittent use reported via these screeners, the provider may use a brief intervention, a screening outcome-responsive conversation that focuses on raising awareness of negative effects, a plan to reduce or stop us, and encouragements of strengths to support behavior change. For those with moderate to high and/or more frequent use, a referral to more intensive services may be needed [11].

Despite AAP recommendations and the evidence base for screening and intervention, the use of standardized screening tools is still not widely implemented in adolescent primary care. Patient factors that may impede screening include level of comfort to discuss sensitive topics [18] and concern about confidentiality [19]. For providers, barriers include time constraints, feeling less capable of making a diagnosis, disagreements on who should implement screening tools, perception of difficulty in discussing substance use, and doubt regarding effectiveness of intervention [20, 21]. Evidence suggests that pediatric primary care providers who reported feeling prepared to diagnose substance use disorders have higher levels of screening [22]. Additional training and resource support may help providers implement brief interventions with or without referral to treatment [2, 20].

The purpose of this study was to describe the distribution and characteristics of substance use screening among adolescents in the outpatient clinics of a large academic medical center in the Midwest (i.e., e.g., which substances screened, how screened) as the first step in the process of identify and addressing substance use problems, to assess patient factors that may be associated with screening (i.e., patient age, race/ethnicity, gender, and insurance type), and evaluate the documentation of screening in the medical records to inform integration of high quality screening into the routine practice.

## Methods

This study involved a retrospective chart review of unique patient medical records at a large academic children’s hospital in the Midwest. The Institutional Review Board (IRB) at the local site granted an exemption from IRB review, given abstraction and analysis of de-identified data.

### Sample selection

We restricted the sampling frame to cases pertaining to well-child visits for patients ages 12–17, in calendar year 2017 at the three outpatient clinics of the hospital with the largest number of adolescent patients (duplicates were eliminated by including only the first visit within the observation period for patients with multiple visits). We selected 10% of cases by computer-generated random number and de-identified these records for further analysis. The sample size for analysis was based on multiple logistic regression analysis with up to five independent variables [23].

### Measurement and coding

Discrete closed-ended values abstracted from the medical record included age (aged 12–17), race/ethnicity, gender, primary source of insurance, and substance use diagnostic code. Open-ended notes abstracted from the medical record included all elements of social history, history of present illness (HPI), and assessment plan. The entire social history and HPI notes were abstracted for thoroughness, that is, to capture all notes that might include substance use screening information for further coding and analysis (not for analysis of social history and HPI per se, as cases include only well-child visits). Open-ended narrative fields were coded by two separate coders (BR, KG) to classify cases as screened (or not screened) for each substance (alcohol, marijuana, tobacco, other substances); record how they were screened (i.e., clinical approach; assessment of quantity, frequency, problem behavior); whether or not they screened positive for use and, if positive, the assessment plan (including counseling by the provider by type of counseling, and/or referral for further assessment and treatment). Discrepancies in coding were resolved by discussion and consensus. Coded data were compared to AAP recommendations for universal screening of all adolescents ages 12–17 (i.e., 100%) and use of structured and validated screening tools.

### Statistical analysis

SPSS version 25 was used for all analyses. We describe sample characteristics and screening of substance use among patients using measures of frequency, central tendency and dispersion. Given prior associations of demographic factors with provider screening behavior, we regressed socio-demographic factors on the screening variable (i.e., screened for use of alcohol, marijuana, tobacco, and/or other substances) in multiple variable logistic regression models (*p* < .05). Dummy variables were created for race/ethnicity (racial ethnic minority vs. all others), gender (female vs. all others), and insurance status (Medicaid recipient vs. all others) for analysis in the logistic regression model based on patterns of association with the outcome of interest.

## Results

A total of 1270 eligible records were included in the sampling frame, from which we drew a sample of 127 cases (10%) for analysis. In terms of socio demographic characteristics (Table 1), the mean and modal age was 14 (SD = 1.44; Note there were no patients aged 17 years). Most patients (76%) identified as Black or Latino in terms of primary race/ethnicity, and most were male (54%) in terms of sex. Primary insurance status was listed most often as Medicaid or Medicaid Managed Care.

**Table 1:** Sample characteristics of adolescent patients (*N* = 127)

	n (%)
Age	
12	**29 (23)**
13	**18 (14)**
14	**32 (25)**
15	**25 (20)**
16	**23 (18)**
17	**0 (0)**
Race/ethnicity	
Black	**48 (38)**
Latinx	**48 (38)**
White	**17 (13)**
Asian	**9 (7)**
Other	**5 (4)**
Gender	
Female	**59 (47)**
Male	**68 (53)**
Insurance type	
Medicaid or Medicaid managed care	**114 (90)**
Private	**11 (9)**
Self-pay	**2 (1)**

Rates of screening (Table 2) were 72% for alcohol, marijuana and tobacco (each substance), and 66% for other substances. All screening was completed using clinical mnemonic cues (e.g., HEEADSSS, SSHADESS). A total of 6% of screened patients reported alcohol use and 6% marijuana use, and only 1 patient reported smoking (i.e., reporting vaping, but type of substance not defined); none reported other substance use. In the vast majority of cases (92%), patients who reported substance use were provided with either anticipatory guidance or counseled using brief motivational strategies to reduce use, with an even split between these who modes of intervention. A total of 30% of those who reported substance use received a referral to intensive services (e.g., mental health counseling, a combination of mental health and substance use counseling, or a general referral to the social worker). There was no documentation of the use of formal substance use screening tools with any of the patients (e.g., CRAFFT) and no consistent pattern for assessment of the frequency, quantity and problem behavior associated with substance use among patients reporting use. All patients who reported substance use were asked about either the frequency or quantity of use, but only two patients were asked both, and those two patients were asked about problem behavior, as well. Factors associated with screening for any substance in the multiple logistic regression model included older age and racial/ethnic minority status (Table 3).

**Table 2:** Distribution of Screening and Reported Use

Substance	Screened *N* (%)	Report use *N* (%)
Alcohol	91 (72)	8 (6)
Marijuana	91 (72)	8 (6)
Tobacco	92 (72)	1 (1)
Other drugs	84 (66)	0 (0)

**Table 3:** Multiple logistic regression on completed screening for any substance*

Factors	*p*	Odds Ratio	95% CI for Odds Ratio	
			Lower	Upper
Age	.001	9.40	3.93	22.44
Race/ethnic minority	.004	39.80	3.25	486.74
Female	.097	3.15	.81	12.23
Medicaid	.580	1.78	.23	13.53

## Discussion

While evidence suggests that provider screening of adolescents for substance use in primary care may be increasing in recent years, which may be attributable to a response to local and national education efforts, [24] the 72% rate of screening for key substances use found in this study is not optimal, and may result in missed opportunities to address substance use for almost 30% of patients. As we note in the introduction, prior literature suggests that time constraints, difficulty in discussing substance use, and doubt regarding effectiveness of intervention, among other factors, are barriers to screening among providers [20, 21]. Even after a recent effort in one state (Massachusetts) to distribute a screening, brief intervention and referral toolkit to adolescent providers across the state, which raised the level of screening to more than 95%, the use of validated tools was only 56% [24]. Lack of time and staff resources were reported as persistent barriers to screening.

We found in our study that providers inconsistently asked about key aspects of substance use, such as the frequency, quantity of consumption and problem behavior that form part of structured screening tools. Thus, although a young person may report that they only drink “once a week” the amount consumed in that episode may be quite high and reflect problematic use that would go undetected if only frequency of use is asked. In addition, we found that patients in this sample reported use of alcohol and marijuana at very low rates (6%), when compared to national samples of high school age youth. This may be the result of social desirability bias in the clinician-patient encounter. This low rate of report is more akin to reports of recent (last month) use by high school age youth [1]. Thus, standardized screening may reduce the potential for recency bias, identify youth who use substances intermittently, and improve consistency and quality of screening overall.

Finally, the association of screening in this study with older patient age is consistent with a prior large, nationally-representative study of adolescent primary care providers conducted more than 15 years ago [25]. We also found that providers tended to screen racial/ethnic minority youth more often than White youth, which is consistent with findings from a study of adult alcohol screening in primary care from the Behavioral Risk Factor Surveillance System (BRFSS) more than 15 years ago [26]. These results highlights the need for continued education efforts among providers; lack of standardized screening may result in failure to adequately screen all adolescents, regardless of age or race/ethnicity.

### Implications

Study results have informed the approach to screening at our outpatient primary care clinics. Given the persistent association of time and staff resources with provider screening behavior, we are building screening tools into our electronic medical record with the intention of deploying the screener on at least an annual basis to all adolescents seen in the primary care setting. Deploying this screening tool in the waiting room, prior to clinical visit, on a smartphone or tablet, with automated calculation of the screening score will save provider time. The implementation of the standard screener will also facilitate interaction with the patient with an option to review or adjust answers during the clinical visit or assist the adolescent in completing the screener if needed. Given the importance of provider education and confidence regarding substance use intervention, we will also conduct additional training around using and interpreting the screening tool, as well as training on providing brief interventions for positive screens. For those adolescents who may benefit from referral to treatment, we have designed an adjunctive substance use program and related resources, including a referral database to share with providers and families. Of note, for other practices that may not have the time or resources to build screening tools into their electronic medical record, paper and electronic versions of some of the validated screening tools are available online.

#### Limitations

Results from this study are limited by the inclusion of a small number of outpatient clinics at one academic children’s hospital and relatively small sample size, thus findings may not generalize to other clinics or hospital settings. Patients in this setting were almost evenly split in terms of male and female gender, were diverse in terms of race and ethnicity (87% non-White), and largely publicly insured, thus findings may not generalize to other, less diverse populations. While the sample was largely diverse, we did not have 17-year-old patients in our sample. This may be because well child visits taper at this age and thus, not including both sick and well-child visits may have limited inclusion of this group. We did not collect information on individual services by provider and therefore were not able to link specific screening practices to provider characteristics. Provider characteristics, such as experience and training in substance use, in particular, may influence screening practices. In addition, based on the limitations of data abstracted from medical records, we were unable to determine why providers did or did not screen, thus obstacles for specific providers are not known. The records reviewed in this study did not document whether or not parents were in the room when screening was completed, thus the low rate of positive screens may be due to lack of confidentiality.

## Conclusions

In summary, despite the AAP recommendation for routine screening of substance use among adolescents in primary care, evidence from this study suggests that low rates of screening by providers persist and may also be related to and race/ethnicity. Not using a standardized screening tool may result in failure to detect important characteristics of use, such as frequency, quantity and problem behavior that indicate problem use. We provide one example of a hospital-level self-examination and the incorporation of universal, automated and structured screening in response to findings. We hope this will encourage and promote other health care institutions to do the same.

## Data Availability

Not applicable.

## Abbreviations

AAP: American Academy of Pediatrics
BSTAD: Brief Screener for Tobacco, Alcohol and Other Drugs (BSTAD)
S2BI: Screening to Brief Intervention (S2BI)
HPI: History of Present Illness
IRB: Institutional Review Board
SAMHSA: Substance Abuse and Mental Health Services Administration
SBIRT: Substance Use Screening, Brief Intervention, and Referral to Treatment
US: United States
USPSTF: United States Preventative Services Task Force
